# 

**Published:** 1826-07

**Authors:** 


### MEDICO-CHIRURGICAL

### REVIEW.

103626 W...
727

*NEW SERIES.*

---

### VOLUME FIVE.

---

[BEING VOL. IX. OF ANALYTICAL SERIES.]

CONDUCTED BY

ASSOCIATED PHYSICIANS AND SURGEONS;

AND SUPERINTENDED BY

JAMES JOHNSON, M.D.

MEMBER OF THE ROYAL COLLEGE OF PHYSICIANS OF LONDON, AND PHYSICIAN EXTRAORDINARY TO HIS ROYAL HIGHNESS THE DUKE OF CLARENCE.

---

Nec tibi quid liceat sed quid fecisse decebit
Occurrat mentemque domat respectus honesti.—CLAUD.

---

Surgeon Gen.'s Office
LIBRARY
57444
Washington, D.C.

LONDON:

*Printed by G. Hayden, Little College Street, Westminster.*

Published by Burgess & Hill, Windmill Street, Haymarket; Baldwin, Cradock, and Joy, Pater-Noster Row; Kingsbury, Parbury, & Allen, Leadenhall Street; Highley, and T. & G. Underwood, Fleet Street; Callow & Wilson, Princes Street, Soho; Cox & Son, Borough; Jackson, Borough; Anderson, West Smithfield; John Nimmo, Great Maze Pond, Borough; Adam Black, Edinburgh; D. Lizars, Princes Street, Edinburgh; Richard Griffin & Co., W. R. M'Phun, and David Allan & Co. Glasgow; Hodges & M'Arthur, Dublin; Messrs. Treuttel & Wurtz, Paris and Strasburg.

---

1826.

W1
ME 75 T

### XII.
### BIBLIOGRAPHICAL RECORD;

OR,

*Works received for Review between the 15th of March and the 15th of June, 1826.*

1. A Letter addressed to the Medical Profession, on the Encroachments on the Practice of the Surgeon-Apothecary by a New Set of Physicians. By Medico-chirurgus. 8vo. sewed, pp. 20. Anderson, London, March, 1826.

☞ *See Periscope, page, 209.*

---

2. Transactions of the Medical and Physical Society of Calcutta. Volume the First. 8vo. pp. 410. Calcutta, May, 1825.

☞ *Some notice of this interesting stranger will be seen at page 36, This volume contains 33 Articles, besides an Appendix of minor papers and intelligence. Almost the whole of the papers are of interest to the profession in this country, as well as to our brethren in the East.*

---

3. Revue Medicale. Feb. Mars. Avr. Mai.

☞ *In Exchange.*

---

4. Philadelphia Journal of the Medical and Physical Sciences, No. 4. New Series, 1826. Conducted by Drs. Chapman, Dewees and Godman.

☞ *In Exchange.*

---

5. The Medical Recorder of Original Papers and Intelligence in Medicine and Surgery. Conducted by Samuel Colhoun, M.D. &c. &c. No. 33, Quarterly Series, for January, 1826. 8vo. pp. 224. Philadelphia, 1826.

☞ *In Exchange.*

---

6. The Edinburgh Journal of Medical Science, No. 2, April, 1826.

☞ *In Exchange.*

---

7. A Practical Treatise on Diabetes: with Observations on the Tabes Diuretica, or Urinary Consumption, especially as it occurs in Children; and on Urinary Fluxes in general. With an Appendix of Dissections and Cases, illustrative of a Successful Mode of Treatment: and a Postcript of Practical Directions for Examining the Urine in these Diseases. By Robert Venables, M.D. Physician to the Henley Dispensary, &c. &c. &c. London, 8vo. pp. 214, 1825.

---

8. A Practical Dissertation on the Means of Obviating and Treating the Varieties of Costiveness, which occur at different Periods of Life, and in Cases of Predispositions to various Constitutional Maladies, in peculiar Temperaments of Body, in Disorders of the Lungs, Stomach, Liver, Rectum, &c. and during Pregnancy, by Medicine, Diet, &c. By Richard Reece, M.D. Fellow of the Royal College of Surgeons, &c. London, 8vo. pp. 351, 1826. Longmans'.

9. The Anatomy of the Brain, with a General View of the Nervous System. By G. Spurzheim, M.D. of the Universities of Vienna and Paris; Licentiate of the Royal College of Physicians in London. Translated from the unpublished French M.S. By. R. Willis, Member of the Royal College of Surgeons in London. With Eleven Plates. London, 8vo. pp. 234. 1826.

---

10. Observations on the Prevailing Practice of Supplying Medical Assistance to the Poor, commonly called the Farming of Parishes; with Suggestions for the Establishment of Parochial Medicine Chests; or Infirmaries in Agricultural Districts. The profits arising from the sale of this Publication will be applied to the benefit of the Eye and Ear Infirmary, established at Southam, 13th April, 1818. London, 8vo, pp. 32—15. 1819.

---

11. An Essay on Headachs, and on their Cure. By Walter Vaughan, M.D. of the Royal College of Physicians in London. 8vo. pp. 252. London, 1825.

---

12. An Account of the Morbid Appearances Exhibited on Dissection in various Disorders of the Brain; with Pathological Observations, to which a Comparison of the Symptoms with the Morbid Changes has given rise. By Thomas Mills, M.D. Licentiate of the King and Queen's College of Physicians. Dublin, 8vo. pp. 238. 1826.

---

13. An Inquiry Concerning that Disturbed State of the Vital Functions usually denominated Constitutional Irritation. By Benjamin Travers, F.R.S. Senior Surgeon to St. Thomas's Hospital, &c. &c. &c. London, 8vo. pp. 556. 1826.

☞ *See present Number.*

---

14. Practical Observations on the Convulsions of Infants. By John North, Surgeon Accoucheur, Member of the Royal College of Surgeons. London, 8vo. pp. 282, 1826. Price 7s. 6d.

☞ *See present Number.*

---

15. Observations on M. Laennec's Method of Forming a Diagnosis of the Diseases of the Chest by means of the Stethoscope and Percussion; and upon some Points of the French Practice of Medicine. By Charles Scudamore, M.D. F.R S. Member of the College of Physicians in London. London, 8vo. pp. 123, 1826.

---

16. The Medical Evidence relative to the Duration of Human Pregnancy, as given in the Gardner Peerage Cause, before the Committee for Privileges of the House of Lords in 1825-6. With Introductory Remarks and Notes. By Robert Lyall, M.D. F.L.S. &c. &c. &c. London, 8vo. stitched, pp. 104. 1826. 4s. 6d.

---

17. Annual Report of the National Vaccine Board to the Secretary of State, &c. and on other Papers relative to Vaccination, from the 1st December, 1825. Folio, pp. 13. March, 1826.

---

18. A Case of Melanosis, with General Observations on the Pathology of this interesting Disease. By Thomas Fawdington, Member of the Royal College of Surgeons, London, and one of the Surgeons to the Manchester Lying-in Hospital. Illustrated by coloured Lithographic Plates. London, 8vo. pp. 49. 1826 7s. 6d.

The following Works have been received through M. J. B. BAILLIERE, Bookseller, Rue de l'Ecole de Medecine, Paris, and No. 3, Bedford-street, Bedford-square, London.

19. Du Magnétisme Animal en France, et des Jugements qu'en ont portés les Societés Savantes, avec le Texte des divers Rapports faits en 1784, par les Commissaires de l' Académie des Sciences, de la Faculté et de la Société Royale de Médecine, et une Analyse des dernières Séances de l' Académie Royale de Médecine et du Rapport de M. HUSSON; suivi de Considérations sur l' Apparition de l' Extase, dans les Traitemens Magnétiques. Par ALEXANDRE BERTRAND, Ancien Elève de l'Ecole Polytecknique, Docteur en Médecine de la Faculté de Paris, &c. 8vo. pp. 539. J. B. Baillière, Paris and London, 1826. 7s.

---

20. Pyrétologie Physiologique, ou Traité des Fièvres Considerées dans l' Esprit de la Nouvelle Doctrine Médicale; Par F. G. BOISSEAU, Docteur en Médecine de la Faculté de Paris, &c. &c. &c. Troisième Edition. 8vo. pp. 722. Baillière, Paris and London, 1826. 9s.

---

21. Exposé des divers Procédés Employés jusqu' à ce Jour pour Guérir de la Pierre, sans avoir recours à l' Opération de la Saille; Par J. LEROY (d' Etiolle,) Docteur en Médecine. 8vo. pp. 232, Four Plates. Baillière, Paris and London, 1825. 4s.

---

22. Anatomie Pathologique. Dernier Cours de Xavier Bichât, d'après un Manuscrit Autographic de P. A. Béclard; avec une Notice sur la Vie et les Travaux de Bichât. Par F. G. BOISSEAU, Membre des Académies Royales de Médecine de Paris et de Madrid, &c. 8vo. pp. 329, with a Plate of Bichât. Baillière, Paris and London, 1825. 5s.

---

23. Traité Clinique et Physiologique de l'Encéphalite, ou Inflammation du Cerveau, et des Suites, telles que le Ramollissement, la Suppuration, les Abcés, les Tubercules, le Squirre, le Cancer, &c. Par M. J. BOUILLAUD, Docteur en Médecine de la Faculté de Paris, &c. &c. 8vo. pp. 350, Baillière, Paris, 1825. 6s.

---

24. Traité des Maladies du Cœur et des Gros Vaisseaux. Par R. J. BERTIN, Professeur d'Hygiéne à la Faculté de Médecine de Paris, &c. &c. &c. Rédigé par J. BOUILLAUD, Docteur en Médecine de la Faculté de Paris, &c. Avec six Planches. 8vo. pp. 464. Bailliére, Paris and London, 1824. 7s.

☞ *We shall shortly review this important work.*

---

25. An Essay on Cupping, &c. &c. By CHARLES KENNEDY, Surgeon, &c. &c. 12mo. pp. 63, with a Plate. London, 1826.

---

26. Dissertatio Medica Inauguralis de Ciborum Concoctione Læsâ. Auctore THOMAS HUGHES, Cambro Britanno, Coll. Reg. Chir. Edin. Socio. Edinburgi, 1825.

---

27. A Picturesque and Topographical Account of Cheltenham, and its Vicinity. By the Rev. T. D. FOSBROKE, M.A. F.A.S. Honorary Associate of the Royal Society of Literature, &c. To which are added contributions towards the Medical Topography, including the Medical History of the Waters. By JOHN FOSBROKE, Resident Surgeon at Cheltenham. Cheltenham, 8vo. pp. 318, with a Map, 1826.

28. Directions for Drinking the Cheltenham Waters; with a Selection of Cases, illustrating their Effects in a great Variety of Diseases. By James M'Cabe, M.D. Author of "Observations on the Cheltenham Waters," &c. &c. Cheltenham, 12mo. pp. 68.

29. An Exposition of the State of the Medical Profession in the British Dominions; and of the Injurious Effects of the Monopoly by Usurpation of the Royal College of Physicians in London. London, 8vo. pp. 373, 1826. Price 9s.

30. Considerations Pratiques sur Certaines Affections de L'Uterus, en Particulier sur la Phlegmasie Chronique avec Engorgement du Col de cet Organe, et sur les Avantages de l'Application Immediate des Sangsues, methodiquement employée dans cette Maladie. Par J. N. Guilbert, M.D. &c. &c. &c. 8vo. Paris, 1826.

☞ *We shall give an analysis of this interesting little work of the learned Guilbert in our next Number.*

31. The Book of Nature. By John Mason Good, M.D. &c. &c. In three volumes Octavo, London, 1826. Price 1*l.* 15s. Longman and Co.

☞ *These volumes contain a Series of Lectures delivered at the Surrey Institution, some years ago, by the learned author. They treat, (as may be supposed from the title,) of*

——————— the great globe itself
And all which it inhabit.

32. Farther Remarks on Hernia, in explanation of the Nature of Strangulation, and of Obliterated Intestine, and in Defence of Views and Suggestions towards Improvement in the Treatment. By E. Geoghegan, M.R.C.S. &c. in a Letter to John Abernethy, Esq. 8vo. stitched, pp. 23. Dublin, 1826.

33. A Corrected Report of the Speeches delivered by Mr. Lawrence, as Chairman at two Meetings of Members of the Royal College of Surgeons, held at the Freemason's Tavern. With an Appendix, containing the Resolutions agreed to at the First Meeting, and some Illustrative Documents. Published at the request of the Committee appointed at the First Meeting. London, 8vo. pp. 133. 1826.

34. The Surgeon-Dentist's Anatomical and Physiological Manual. By G. Waite, Member of the Royal College of Surgeons. London, 12mo. pp. 214. 1826.

35. Practical Observations in Surgery: more particularly as regards the Naval and Military Service. Illustrated by Cases, and various Official Documents. Second Edition, considerably enlarged. By Alex. Copland Hutchison, late Surgeon to the Royal Naval Hospital at Deal, &c. &c. &c. London, 8vo. pp. 422, with a Plate. 1826.

☞ *We hope to be able to notice this interesting work in our Next.*

36. The North American Medical and Surgical Journal. Conducted by Drs. Hodge, Bache, Meigs, Coates and La Roche. No. 11, April, 1826. Philadelphia.

37. The American Medical Review, and Journal of Original and Selected Papers in Medicine, and Surgery. Vol. II. Nos. 1, 2, for September and December, 1825. Philadelphia.

☞ *In Exchange.*

38. A Treatise on the Physiology and Diseases of the Ear; containing a Comparative View of its Structure and Functions, and of its Various Diseases, arranged according to the Anatomy of the Organ, or as they affect the External, the Intermediate and the Internal Ear. Fourth Edition; with considerable Additions and Improvements. By JOHN HARRISON CURTIS, Esq. Aurist to His Majesty, &c. &c. London, 8vo. pp. 236, with a Plate. 1826.

---

39. A Catechism of Anatomy; for the Instruction of Youth in the First Principles of that Science. Duodecimo, pp. 72. Whittaker, June, 1826, Price 9 pence.

---

40. Experimental Researches on the Influence exercised by Atmospheric Pressure upon the Progression of the Blood in the Veins, upon that Function called Absorption, and upon the Prevention and Cure of the Symptoms Caused by the Bites of Rabid or Venomous Animals. (Dedicated by Permission to His Majesty.) With an Appendix containing the Original Reports of Baron Cuvier, and Professors Dumeril and Laennec, to the Royal Academy of Medicine of Paris, &c. &c. By DAVID BARRY, M.D. &c. &c. London, 8vo. pp. 175, with a Lithographic Plate, 1826.

---

41. An Introduction to the Study of the Laws of Chemical Combination and the Atomic Theory. Drawn up for the Use of Students by EDWARD TURNER, M.D. F.R.S.E. Lecturer on Chemistry, and Fellow of the Royal College of Physicians, Edinburgh. Edinburgh, 12mo. pp. 114. 1826.

---

42. A Treatise on the Effects and Properties of Cold, with a Sketch, Historical and Medical, of the Russian Campaign. By MORICHEAU BEAUPRÉ, M.D. Regimental Surgeon in the French Service. Translated by JOHN CLENDINNING, A.B. & M.D. with an Appendix by the Translator. Edinburgh, 8vo. pp. 375. 1826.

---

43. A. Corn. Celsi Medicinæ libri octo ex recensione Leonardi Targæ. Quibus accedunt Tituli Marginales perpetui, capitum librorumque; Annotationes Criticæ, Medicæ, Physicæ: Tabulæ characterum, ponderum, mensurarum, aliæ; Indices Materiæ Medicæ Celsianæ, Rerumque, Omniumque Locupletissimi: Prefixa de Celsi vitâ Dissertatione Concinnavit EDWARDUS MILLIGAN, M.D. S. A.S.S. Collegii Regii Medicorum Edinburgensis Sodalis, &c. &c. &c. Edinburgi, 8vo. pp. 483. 1826.

☞ *In all that regards Celsus, Dr. Milligan has given full vent to his enthusiasm for that author; and to a text of immaculate purity, has made many extensive additions. We find many of the hitherto inexplicable passages illustrated; the text commodiously subdivided, and rendered accessible for reading, or reference by side-titles, and indexes. The great difficulty which had to be surmounted, was the establishment of the identity of the diseases, medicinal substances and remedies discussed by Celsus, with the individuals of modern nomenclature, and it was scarcely a lighter task to fix, as the Doctor has done beyond all doubt, the relations of the weights and measures he employs to those at present prescribed by physicians. The characters used in abbreviations for these weights by the ancients, were also to be rediscovered from the rare, and almost inaccessible works, over which they were scattered: and we find that in his researches, Dr. Milligan has laid down the original sources of his conclusions with sufficient precision, to enable the merest tyro to satisfy himself of their legitimacy. By the help of a synopsis, with the numerous tables and indexes, Celsus may now be perused with the same facility*

*and profit as Heberden or Sydenham. We need hardly, after this, say that Dr. Milligan's edition of Celsus ought to become a classical and standard work in the library of every medical man who wishes to be acquainted with this elegant ancient writer and most admirable compiler of all the medical knowledge that had accumulated up to his time.*

---

*** NOTE.—Authors and publishers will readily appreciate the importance of having their works recorded, with full length titles, on this list, which stands as a perpetual advertisement so long as the Journal lasts, and as far as it extends. The republication of the Journal in America enhances the advantages of the BIBLIOGRAPHICAL RECORD, on which no work can be entered, unless transmitted, free of expense, to the Editor, under sealed cover to the publishers, or in any other way most convenient to the parties concerned.

*N. B.—As the Bibliographical Record closes on the* 15*th of the month preceding publication, all Works received after that date necessarily stand over till next quarter.*

---

### XIII.

### MEDICAL MISCELLANY.

### *Additional Subscribers since last Quarter.*

A.

Archer, Mr. William, East India Company's Service.

B.

Bouchier, Thomas, Esq. Surgeon, 36th Regiment.

Burrows, Mr. E. Surgeon, 114, High Holborn.

C.

Callow, Mr. Surgeon, 81st Regt.

Campbell, William, Esq. Argyll-street.

Carrol, James, F. M.D. Limerick.

Creed, George, Esq. Surgeon, West Suffolk Militia, Bury St. Edmunds.

Corbyn, Mr. East Indies.

Cornelius, M. Hamburgh.

E.

Edmonston, Henry, M.B. New-castle upon Tyne.

H.

Heron, Mr. Surgeon, Charlotte-street, Fitzroy-square.

I.

Ives, Thos. Esq. Surgeon, Cholham, Surrey.

Jenkins, Mr. Surgeon, Gosport.

M.

Mason and Scarwell, St. Osyth.

M'Dermott, John, M.D. Ayre.

M'Dowall, Mr. J. Surg. Glasgow.

Miller, Mr. Chas. R.N. M.R.C. Hastings.

Morgan, Mr. Surgeon, Sloane-street.

N.

Nevison, Mr. Assistant Surgeon, 5th Regiment.

O.

Osmond, Mr. Surg. Thorpe.

P.

Parkinson, Dr. Woolwich.

Parrott, William, Esq. Surgeon, Cape Corps.

R.

Rhood, Mr. Surgeon.

Robertson, Mr. Assistant Surgeon, 70th Regiment.

S.

Scott, Mr. Surgeon, Newington Causeway, Borough.

W.

Watson, Dr. Staff Surgeon, Portsmouth.

Williams, Mr. Assistant Surgeon, 27th Regiment.

Wilson, James, Esq. to Buenos Ayres.

Wilton, Dr. Upminster, Essex.

Y.

Young, Mr. Surgeon.

---

A practice or a partnership is wanted by a gentleman who is properly qualified, and who is well known to the Editor of this Journal. Application (post paid) or verbally may be made to Dr. Johnson.

☞ No. 8 of this Journal, may now be had by application through the proper channels.

Owing to a temporary illness of the Editor (from which he is now recovered) some typographical errors have crept into this Number, while passing through the press. A more particular corrigenda will be given at the end of the next Number, which concludes a volume.

p. 46—for *mummication* read *mummification*.

51—for *psamureticus* read *psammeticus*.

London; May 30, 1826.

THE

### London Medical and Physical Journal,

EDITED BY DR. MACLEOD.

---

**For many fortunate discoveries in medicine, and for the detection of numerous errors, the world is indebted to the rapid circulation of Monthly Journals; and there never existed any work, to which the Faculty, in Europe and America, were under deeper obligations, than to the Medical and Physical Journal of London, now forming a long, but an invaluable, series.—RUSH.**

---

The Proprietors of this Journal beg to inform the Medical Profession, and the Public, that they intend to commence a New Series on the 1st of July. They have had this measure in contemplation for some time, in consequence of frequent applications for an opening, having been made to them by gentlemen desirous of becoming subscribers.

The circumstance, however, which has decided them in choosing the present period for carrying the measure into effect, is their desire to bring fully before the Profession certain improvements, which they trust will be regarded as essentially enhancing the value of their publication. These relate both to the matter and the manner in which it will be presented to the reader.

1st. An *entirely new Department* will be added, consisting of instructive CASES obtained from PUBLIC INSTITUTIONS, and other authentic sources. These, they have reason to believe, will constitute a mass of medical and surgical information, greatly exceeding in importance any thing of the kind which they have hitherto been able to procure.

2dly. The typography of the Journal will be much improved, as they have obtained NEW TYPES expressly for the purpose; while, in order to meet the additional quantity of matter, *eight pages* will be added to each Number; being an addition of *forty-eight pages* to the volume.

For the spirit in which the Work will be conducted, the Proprietors take leave to refer to the last few volumes. They have the best reasons for believing that the labours of the present Editors have met the approval of their professional brethren; reasons founded on the increase in the number and value of the Original Communications, as well as the extended sale of the Journal.

In other respects, no essential change will take place: the ORIGINAL PAPERS will be rendered still more select;—the CRITICAL ANALYSES of all important Works, both domestic and foreign, will be continued;—and the departments of COLLECTANEA and INTELLIGENCE will, as before, contain short notices of cases, and facts of every kind interesting to the Medical Profession, arranged according to subjects.

The *new Series* will constitute an immediate continuation of the *old;* the numbering of which will be continued, as well as that of the Series about to be commenced. The Journal will thus form a complete record of Medical Literature, from the beginning of the present century.

Orders for the New Series will be received by all Booksellers; and those who intend to become Subscribers are respectfully requested to intimate their intentions as soon as possible, in order that the additional number of copies struck off may be made as nearly as possible to correspond with the demand.

The Proprietors beg to add, that, notwithstanding the very considerable expense incurred by these new arrangements, the price of the Journal will continue the same as before, viz. 2s. 6d. per Number.

ADVERTISEMENTS will be inserted on the Cover, and BILLS stitched in the Work, as hitherto, at the usual charges.

---

Published by J. SOUTER, 73, St. Paul's Church Yard; and may be had through the medium of all Booksellers, in Town and Country.

Printed by J. and C. Adlard, Bartholomew Close.

